# Healthy Eating and Risks of Total and Cause-Specific Death among Low-Income Populations of African-Americans and Other Adults in the Southeastern United States: A Prospective Cohort Study

**DOI:** 10.1371/journal.pmed.1001830

**Published:** 2015-05-26

**Authors:** Danxia Yu, Jennifer Sonderman, Maciej S. Buchowski, Joseph K. McLaughlin, Xiao-Ou Shu, Mark Steinwandel, Lisa B. Signorello, Xianglan Zhang, Margaret K. Hargreaves, William J. Blot, Wei Zheng

**Affiliations:** 1 Division of Epidemiology, Vanderbilt University School of Medicine, Nashville, Tennessee, United States of America; 2 International Epidemiology Institute, Rockville, Maryland, United States of America; 3 Department of Medicine, Vanderbilt University School of Medicine, Nashville, Tennessee, United States of America; 4 Vanderbilt-Ingram Cancer Center, Vanderbilt University, Nashville, Tennessee, United States of America; 5 Department of Epidemiology, Harvard School of Public Health, Boston, Massachusetts, United States of America; 6 Department of Internal Medicine, Meharry Medical College, Nashville, Tennessee, United States of America; University of Oxford, UNITED KINGDOM

## Abstract

**Background:**

A healthy diet, as defined by the US Dietary Guidelines for Americans (DGA), has been associated with lower morbidity and mortality from major chronic diseases in studies conducted in predominantly non-Hispanic white individuals. It is unknown whether this association can be extrapolated to African-Americans and low-income populations.

**Methods and Findings:**

We examined the associations of adherence to the DGA with total and cause-specific mortality in the Southern Community Cohort Study, a prospective study that recruited 84,735 American adults, aged 40–79 y, from 12 southeastern US states during 2002–2009, mostly through community health centers that serve low-income populations. The present analysis included 50,434 African-Americans, 24,054 white individuals, and 3,084 individuals of other racial/ethnic groups, among whom 42,759 participants had an annual household income less than US$15,000. Usual dietary intakes were assessed using a validated food frequency questionnaire at baseline. Adherence to the DGA was measured by the Healthy Eating Index (HEI), 2010 and 2005 editions (HEI-2010 and HEI-2005, respectively). During a mean follow-up of 6.2 y, 6,906 deaths were identified, including 2,244 from cardiovascular disease, 1,794 from cancer, and 2,550 from other diseases. A higher HEI-2010 score was associated with lower risks of disease death, with adjusted hazard ratios (HRs) of 0.80 (95% CI, 0.73–0.86) for all-disease mortality, 0.81 (95% CI, 0.70–0.94) for cardiovascular disease mortality, 0.81 (95% CI, 0.69–0.95) for cancer mortality, and 0.77 (95% CI, 0.67–0.88) for other disease mortality, when comparing the highest quintile with the lowest (all *p*-values for trend < 0.05). Similar inverse associations between HEI-2010 score and mortality were observed regardless of sex, race, and income (all *p*-values for interaction > 0.50). Several component scores in the HEI-2010, including whole grains, dairy, seafood and plant proteins, and ratio of unsaturated to saturated fatty acids, showed significant inverse associations with total mortality. HEI-2005 score was also associated with lower disease mortality, with a HR of 0.86 (95% CI, 0.79–0.93) when comparing extreme quintiles. Given the observational study design, however, residual confounding cannot be completely ruled out. In addition, future studies are needed to evaluate the generalizability of these findings to African-Americans of other socioeconomic status.

**Conclusions:**

Our results showed, to our knowledge for the first time, that adherence to the DGA was associated with lower total and cause-specific mortality in a low-income population, including a large proportion of African-Americans, living in the southeastern US.

## Abstract 2

In a prospective cohort study, Wei Zheng and colleagues study the association between adherence to dietary guidelines and mortality in low-income US adults, two thirds of whom are African-Americans.

## Editors' Summary

**Background:**

Certain parts of the population, including women, children, ethnic and racial minorities, and poor people, are often underrepresented in clinical trials and in epidemiological studies (which examine the patterns, causes, and effects of health and disease conditions). In the US population, the link between diet and health has mostly been studied in non-Hispanic white individuals from middle- and high-income households. Such studies formed the basis for the Dietary Guidelines for Americans (DGA), and more recently have shown that adherence to the DGA is associated with lower levels of obesity, as well as lower risks for diabetes, cardiovascular disease (such as heart attacks and strokes), and certain cancers. To measure adherence to the DGA, the Center for Nutrition Policy and Promotion at the US Department of Agriculture developed the Healthy Eating Index (HEI) in 1995. The DGA and the HEI have been updated several times, and the HEI-2010—the latest version—reflects the 2010 DGA.

**Why Was This Study Done?:**

Because research participants are often not representative of the entire US population, it is unknown whether the results of many studies are valid for all Americans. To remedy this situation, efforts have been made to recruit participants from previously underrepresented parts of the population and to address important health questions in such groups. For this study, the researchers wanted to examine whether adherence to the DGA was associated with better health outcomes in poor people and African-Americans, consistent with the results in wealthier non-Hispanic white individuals.

**What Did the Researchers Do and Find?:**

The researchers analyzed data from the Southern Community Cohort Study (SCCS). The SCCS was funded by the National Cancer Institute and was initiated in 2001 with the goal of addressing unresolved questions about the causes of cancer and other chronic diseases, as well as reasons for health disparities. The SCCS recruited most of its participants from community health centers in 12 states in the southeastern US. These centers serve predominantly poor and uninsured people, including many African-Americans. Of approximately 85,000 SCCS participants, over two-thirds were African-American, and over half were poor, with an annual household income of less than US$15,000.

For this study, the researchers used a food frequency questionnaire that was designed to capture foods commonly consumed in the southeastern US, and from this calculated HEI-2010 scores for each participant. They also collected other health- and lifestyle-related information. They then followed all participants for whom they had complete information (over 77,000) for a number of years (half of them for over 6.2 years). During that period, 6,906 participants died; including 2,244 from cardiovascular disease, 1,794 from cancer, and 2,550 from other diseases. When the researchers tested for a possible association between HEI-2010 and death (controlling for other relevant factors such as age, weight, exercise, smoking, and the presence of specific chronic diseases), they found that participants with a higher HEI-2010 score (reflecting better adherence to the DGA) had a lower risk of dying in the follow-up period. Participants with the healthiest diet (those in the top one-fifth of HEI-2010 scores) had only about 80% of the risk of death of those with the unhealthiest diets (those in the bottom one-fifth of HEI-2010 scores). This reduction in the risk of death by approximately 20% was true for death from any disease, death from cancer, and death from cardiovascular disease.

**What Do These Findings Mean?:**

The results support the validity of the DGA for healthy eating across the US population. However, the study had some limitations. For example, participants were asked only once—when they first joined the SCCS—about their diet, their household income, and other factors that can change over time, such as exercise habits and diseases they have been diagnosed with. Besides such changes, there could be other factors not captured in the study that might influence the association between diet and death. Despite these uncertainties, the findings suggest that adherence to the DGA is associated with lower total mortality and mortality from cancer or cardiovascular disease in poor US Americans in general, and in low-income African-Americans.

**Additional Information:**

Please access these websites via the online version of this summary at http://dx.doi.org/10.1371/journal.pmed.1001830.

- Information is available online about the Southern Community Cohort Study
- The US Department of Agriculture’s Center for Nutrition Policy and Promotion has information on the Healthy Eating Index, which is based on the Dietary Guidelines for Americans
- The World Health Organization provides information on diet as part of its global strategy for diet, physical activity, and health, as well as a factsheet on healthy diet
- Wikipedia has a page on race and health in the US (note that Wikipedia is a free online encyclopedia that anyone can edit)

## Introduction

The Dietary Guidelines for Americans (DGA) are a centerpiece of nutrition policy and principles for nutrition education in the United States [1]. The goal of the DGA is to promote health and prevent chronic diseases by providing evidence-based nutritional guidance to Americans 2 y of age and above. To assess adherence to the DGA, the Healthy Eating Index (HEI) was developed [2]. The 2005 edition of the HEI (HEI-2005) has been examined in several US studies conducted primarily in non-Hispanic white individuals and in populations with a middle or high socioeconomic status (SES) [3–9]. A higher score, which suggests higher guideline adherence and a better quality diet, has been associated with lower levels of obesity and inflammation [3,4] and lower risks for developing diabetes, cardiovascular disease (CVD), and certain cancers [5–9]. The US Department of Agriculture (USDA) and the Department of Health and Human Services update the DGA and HEI every 5 y to reflect recent nutritional evidence [10]. Compared with the HEI-2005, the 2010 edition of the HEI (HEI-2010) placed more emphasis on food quality. In the HEI-2010, whole grains were given more weight and were separated from refined grains; seafood and plant proteins and the ratio of unsaturated to saturated fatty acids were introduced to encourage healthy choices of protein and fat [11]. Only two studies have recently examined the HEI-2010 in relation to mortality, reporting lower mortality from all causes, CVD, cancer, and chronic liver disease [12,13]. Again, these studies were conducted primarily in non-Hispanic white individuals and in populations with a middle or high SES.

Studies have suggested that race/ethnicity and SES influence food choices and dietary compliance. African-Americans and low-SES adults, especially low-income African-American men [14], have been shown to have more limited access to supermarkets and healthy foods [15], to tend to consume more energy-dense and nutrient-poor foods [16], and to have generally lower diet quality scores [17,18], when compared with white individuals and high-SES adults. These sociodemographic factors may further modify the associations between diet and health outcomes [3,19–21]. To our knowledge, no study has adequately evaluated the association between HEI and disease mortality in African-Americans and low-income adults. It is unclear whether the health benefits of following the DGA can be generalized to these underserved populations who experience disproportionally higher disease burden than their white and higher SES counterparts. The objective of this study is to evaluate adherence to the DGA, measured by the HEI-2010, in association with total and cause-specific mortality among 77,572 men and women participating in the Southern Community Cohort Study (SCCS) and eligible for the current analysis. We also reported the corresponding associations for the HEI-2005 to facilitate comparison with previous literature and examined the associations for individual recommendations in the HEI-2010 to potentially inform nutrition policy and clinical practice. The SCCS was designed to fill a gap in study populations not covered in previous research, by recruiting understudied populations, i.e., African-Americans (two-thirds of the SCCS participants) and adults with an annual household income less than US$15,000 (more than half of the SCCS participants). Thus, our study represents a unique investigation, the findings of which could have significant implications in promoting healthy eating to reduce disease mortality in African-Americans and low-income adults in the US.

## Methods

### Study Population

The SCCS is a prospective cohort study investigating health disparities in the southeastern United States, particularly among African-Americans and low-income individuals. From March 25, 2002, to September 24, 2009, 84,735 adults aged 40–79 y were enrolled from 12 southeastern states. Detailed study design and methods have been published [22] and can be found at http://www.southerncommunitystudy.org. Briefly, 86% of the participants were recruited at community health centers (CHCs), which provide basic health services, mainly to low-income, uninsured people. Baseline in-person interviews were conducted at CHCs using a structured questionnaire to collect detailed information on sociodemographics, anthropometrics, medical history, dietary intakes, and lifestyle factors. The remaining 14% of the participants were recruited via mail from the general population residing in the same areas, using the same questionnaire. The protocol of the SCCS was approved by the institutional review boards of Vanderbilt University Medical Center and Meharry Medical College. All participants provided written informed consent. Access to de-identified data is available to the research community with approval from the SCCS Data and Biospecimen Use Committee. Applications can be submitted through the SCCS Online Request System: https://ors.southerncommunitystudy.org.

### Mortality Ascertainment

The SCCS updated participants’ vital status annually by linkage with the Social Security Administration (SSA) and ascertained the date and cause of death via the National Death Index (NDI), which provides nearly full coverage for deaths occurring within the US. The latest linkage was conducted to ascertain all deaths that occurred in the cohort before January 1, 2012. The SCCS also contacted participants to complete follow-up surveys every 4–5 y. Causes of death were classified by the International Classification of Diseases, 10th Revision (ICD-10). External causes of death (ICD-10 codes beginning with the letter V, W, X, or Y) were not counted in disease mortality. CVD deaths were identified by ICD-10 codes starting with the letter I (i.e., I00–I99), cancer deaths were identified by codes starting with the letter C (i.e., C00–C97), and other deaths were defined as other disease deaths not due to CVD or cancer.

### Dietary Assessment

A food frequency questionnaire (FFQ), designed to capture most of the foods commonly consumed in the southeastern US, was administered at baseline [23]. The SCCS FFQ contained 89 food items. For each food item, participants could choose from nine frequency categories based on their usual intake over the past year, from “never” to “once per week” to “2 or more times per day.” To estimate intake amount of each food item, we used the race- and sex-specific portion size from the National Health and Nutrition Examination Survey (NHANES) and the USDA Continuing Survey of Food Intakes by Individuals, restricted to participants who lived in the South census region, aged 30–84 y, and reported as non-Hispanic white or African-American individuals [24]. Intakes of total energy and nutrients were calculated based on the estimated average portion sizes. The validity of the FFQ was assessed in a study conducted among 275 SCCS participants with repeated 24-h dietary recalls during a 3–5-mo period. The correlation coefficients ranged from 0.59 to 0.83 for macronutrients and from 0.43 to 0.81 for micronutrients [25].

To calculate the HEI, the SCCS FFQ data were linked to the MyPyramid Equivalents Database (version 2.0) [26] to generate the equivalent intake (cup or ounce equivalents per 1,000 kcal) for food groups listed in the DGA. The MyPyramid Equivalents Database is especially helpful for mixed dishes and processed foods because it disaggregates food into ingredients and combines ingredients into DGA-based food groups. Then, the equivalent intake of each food group was scored using HEI-2010 or HEI-2005 standards [10]. Both HEIs comprise 12 components with a total score of 100 points. HEI-2010 recommended increasing consumption of nine components: total fruit, whole fruit, total vegetables, greens and beans, whole grains, dairy, total protein foods, seafood and plant proteins, and the ratio of unsaturated to saturated fatty acids; and decreasing consumption of three components: refined grains, sodium, and calories from solid fats, excessive alcohol, and added sugars. Reference SAS codes for calculating HEI scores were provided by the USDA [27].

### Statistical Analysis

We excluded 6,756 participants who had incomplete FFQs (>10 food items left blank) or reported implausible energy intakes (<600 or >8,000 kcal/d), 389 participants with missing race/ethnicity information, and 18 participants who died within 20 d after the baseline survey. After these exclusions, 77,572 cohort members remained for the current analysis.

Participants were classified by sex-specific quintiles of HEI score. Analyses were performed for men and women separately to take into account gender differences in eating behavior, mortality rate, and disease risk factors; results were then combined by fixed-effect meta-analyses, since no significant heterogeneity was detected. Baseline characteristics were compared among participants in the different quintiles of HEI-2010 score using ANOVA for continuous variables and a χ^2^ test for categorical variables. Hazard ratios (HRs) and 95% confidence intervals (CIs) were estimated using Cox proportional hazards regression with age as the time scale. Follow-up time was censored on the date of death for deceased cohort members and December 31, 2011, the last linkage date with the SSA and NDI, for living cohort members. For those whose vital status was reported as unknown by the SSA and not identified as deceased by the NDI (<10 participants), follow-up was censored at the last contact. The proportional hazards assumption was evaluated using Schoenfeld residuals, and no significant violation was found. Potential confounders were adjusted, including enrollment source (CHC, general population), race/ethnicity (African-American, white, other), education (<12 y, graduated high school, some college, graduated college or more), annual household income (<US$15,000, US$15,000–US$24,999, US$25,000–US$49,999, ≥US$50,000), marital status (currently married, other), medical insurance (yes, no), smoking (never, former, current at <1 pack/day, current at ≥1 pack/day), physical activity (quartiles of metabolic equivalent of task—hours), sitting time (quartiles), total energy intake (quartiles), body mass index (BMI) (<18.5, 18.5–24.9, 25.0–29.9, 30.0–34.9, ≥35 kg/m^2^), menopausal status and use of hormone therapy (for women only), and baseline disease status (yes, no, for each disease) for hypertension, diabetes, hypercholesterolemia, chronic obstructive pulmonary disease (COPD), CVD, cancer, and HIV/AIDS. Where data for a covariate were missing (<2% of the population for each of the study variables included in the analysis), individuals were assigned the most frequent category or the median value in each sex. *p*-Value for trend was obtained by treating median HEI score in each quintile as a continuous variable. Associations between HEI component scores and total mortality were assessed using the same Cox model and covariates as in the analysis for the total score, with and without further adjustment for a standardized HEI score excluding the corresponding component.

Stratified analyses were conducted to evaluate potential interactions of HEI score with sex, race, income, smoking habits, and disease history. For racial/ethnic groups, we present findings for African-Americans versus individuals of white or other race/ethnicity. Results from the white and other racial/ethnic groups were combined because the sample size of the “other” racial/ethnic group was small, and the HEI score—mortality associations were similar between the white and other racial/ethnic groups. *p*-Value for interaction was obtained via the likelihood ratio test with and without the interaction terms (dummy variables of HEI score quintile × stratification). A series of sensitivity analyses were conducted, excluding participants who died in the first 2 y of follow-up (*n* = 1,722), participants who were not recruited from CHCs (*n* = 10,591), participants with uncertain cause of death (*n* = 318), or participants with any missing information (*n* = 5,916). Multiple imputation was also performed to estimate HEI-2010 scores for participants who were excluded due to unreliable dietary data and to treat other missing variables. Competing-risks analysis was further performed for cause-specific mortality. SAS 9.3 was used for statistical analyses (SAS Institute, Cary, North Carolina, US), and two-sided *p* < 0.05 was considered statistically significant.

## Results

The present analysis included 20,648 African-American men, 29,786 African-American women, 9,406 white men, 14,648 white women, and 1,134 men and 1,950 women of other race/ethnicity, accounting for 26.6%, 38.4%, 12.1%, 18.9%, 1.5%, and 2.5% of our study population, respectively. At baseline, the mean HEI-2010 score in the SCCS was 57.8 (standard deviation [SD]: 12.1). Generally, men had a poorer diet than women, as reflected by a lower HEI score (S1 Table). The mean HEI score was similar in African-American men and white men, higher in African-American women than white women, and highest in the “other” racial/ethnic group. Notably, individuals with a household income < US$15,000/y had a mean HEI score 3.3 points (95% CI, 3.2–3.5) lower than those with a household income ≥ US$15,000/y.

In both sexes, participants with a higher HEI score were older, had higher education and income levels, and were more likely to be currently married, covered by medical insurance, and not enrolled from CHCs (Table 1). HEI score was positively associated with BMI, total physical activity, and use of postmenopausal hormone therapy by women. Current smokers, particularly current smokers of ≥1 pack/day, were more likely to have a low HEI score than never smokers. With the exception of COPD and HIV/AIDS, individuals with common chronic diseases at baseline, including hypertension, diabetes, hypercholesterolemia, CVD, and cancer, tended to have a higher HEI score than those who did not have these conditions, suggesting possible dietary changes after disease diagnosis.

**Table 1:** Baseline characteristics by sex-specific quintiles of HEI-2010 scores in the Southern Community Cohort Study, 2002–2009.

Category	Characteristic	Men (*n =* 31,188)			Women (*n =* 46,384)		
		Quintile 1	Quintile 3	Quintile 5	Quintile 1	Quintile 3	Quintile 5
**HEI-2010 score**	Median HEI-2010 score	40.6	54.7	70.6	43.7	59.5	75.7
	Range of HEI-2010 scores	13.7–45.3	51.7–57.7	64.8–95.2	16.9–48.8	56.2–62.6	70.2–96.7
**Sociodemographics**	**Age, years**	50.2 ± 7.8	51.4 ± 8.3	55.3 ± 9.2	49.6 ± 7.7	52.1 ± 8.7	56.0 ± 9.3
	**African-Americans, percent**	63.8	69.1	61.5	59.7	66.8	63.4
	**Education, percent**						
	<12 y	36.0	30.5	20.9	33.3	28.4	19.0
	Graduated high school	42.1	40.8	30.0	42.1	38.8	33.9
	Some college	15.5	17.7	21.1	17.8	20.8	24.8
	Graduated college or higher	6.4	11.0	28.0	6.8	12.0	22.4
	**Annual household income, percent**						
	<US$15,000	62.0	55.8	38.4	62.7	56.3	45.3
	US$15,000–US$24,999	19.6	21.1	18.3	21.8	21.8	21.5
	US$25,000–US$49,999	12.5	13.7	18.5	10.8	14.6	18.8
	≥US$50,000	5.8	9.4	24.9	4.8	7.3	14.4
	**Currently married, percent**	33.4	37.2	50.8	32.6	33.5	34.9
	**Medical insurance covered, percent**	49.5	54.7	70.7	56.6	63.3	70.9
	**CHC enrollment, percent**	89.2	86.6	72.2	90.2	89.2	83.4
**Obesity and lifestyle factors**	**BMI, kg/m** ^**2**^	27.6 ± 6.1	28.1 ± 6.1	28.8 ± 6.2	31.3 ± 8.2	32.0 ± 8.1	31.8 ± 8.2
	**Total physical activity, MET-hours**	23.8 ± 21.8	25.3 ± 21.8	25.0 ± 22.2	20.0 ± 16.0	21.0 ± 15.8	21.7 ± 16.1
	**Total sitting time, hours**	9.2 ± 5.2	9.2 ± 5.1	9.2 ± 5.2	9.5 ± 5.0	9.4 ± 4.9	9.0 ± 5.0
	**Cigarette smoking, percent**						
	Never	18.1	21.1	33.8	36.5	45.0	54.4
	Former	18.1	23.6	37.4	15.0	21.1	28.8
	Current, <1 pack/day	34.4	35.9	20.6	27.2	23.1	12.8
	Current, ≥1 pack/day	29.5	19.5	8.3	21.4	10.8	4.0
	**Postmenopause, percent**				61.0	68.4	78.8
	**Hormone therapy, percent**				24.1	28.6	38.1
**Comorbidities**	**Hypertension, percent**	46.9	49.2	55.1	51.8	58.1	61.1
	**Diabetes, percent**	12.6	18.0	26.8	15.7	23.3	29.9
	**Hypercholesterolemia, percent**	24.0	27.9	41.1	30.2	35.7	44.8
	**CVD, percent**	13.0	13.0	15.7	9.6	11.2	12.2
	**Cancer, percent**	4.7	5.7	9.7	8.7	8.7	10.5
	**COPD, percent**	8.3	6.8	5.1	12.3	10.7	8.2
	**HIV/AIDS, percent**	2.8	2.9	1.9	1.3	0.9	0.5

Data are given as median, range, age-adjusted mean ± SD, or percentage. *p-*Values were all <0.05, except for race and sitting time for men.

MET, metabolic equivalent of task.

During a mean follow-up of 6.2 y (484,744 person-years), we documented 3,672 and 3,234 disease deaths in men and women, respectively. The mortality rates per 1,000 person-years were 19.1 in African-American men, 19.7 in white men, 10.7 in African-American women, and 11.9 in white women. After adjustment for potential confounders, HEI-2010 score showed significant inverse associations with total disease mortality in the total SCCS population and in subpopulations by sex, race/ethnicity, and household income (Table 2). When comparing the highest quintile with the lowest among all SCCS participants, a higher HEI-2010 score was associated with a 20% (95% CI, 14%–27%) lower risk of disease death. The associations were comparable between men and women in analyses that included all participants or by race/ethnicity. African-Americans, on average, had the lowest income level; 59% of them had a household income < US$15,000/y, compared with 46% of white individuals and 49% of individuals of other race/ethnicity. No statistically significant interaction was identified for HEI-2010 score and income level in analyses stratified by race, although the association between HEI-2010 score and mortality was not statistically significant among African-Americans with a household income ≥ US$15,000/y.

**Table 2:** Association of HEI-2010 score and total disease mortality by sex, race, and income in the Southern Community Cohort Study, 2002–2011.

Population	Number of Participants (Deaths)	Person- Years of Follow-Up	Multivariate HR (95% CI) by Quintiles of HEI-2010 Score, Quintile 1 = Referent				*p* for Trend	*p* for Interaction
			Quintile 2	Quintile 3	Quintile 4	Quintile 5		
**Total**	77,572 (6,906)	484,744	0.99 (0.92, 1.06)	0.95 (0.89, 1.03)	0.93 (0.86, 1.00)	0.80 (0.73, 0.86)	<0.001	
Men	31,188 (3,672)	191,531	0.97 (0.88, 1.07)	0.91 (0.83, 1.01)	0.93 (0.84, 1.03)	0.81 (0.72, 0.90)	<0.001	0.76
Women	46,384 (3,234)	293,213	1.01 (0.90, 1.12)	1.00 (0.90, 1.12)	0.93 (0.83, 1.04)	0.78 (0.69, 0.88)	<0.001	
African-American	50,434 (4,614)	328,781	1.00 (0.91, 1.10)	0.98 (0.90, 1.08)	0.94 (0.85, 1.03)	0.81 (0.73, 0.89)	<0.001	0.53
White or other race/ethnicity	27,138 (2,292)	155,963	0.97 (0.86, 1.10)	0.90 (0.79, 1.02)	0.92 (0.81, 1.05)	0.80 (0.69, 0.92)	0.002	
Household income < US$15,000/y	42,759 (4,918)	266,200	0.98 (0.90, 1.07)	0.96 (0.88, 1.05)	0.92 (0.84, 1.01)	0.77 (0.70, 0.85)	<0.001	0.95
Household income ≥ US$15,000/y	34,813 (1,988)	218,544	0.99 (0.85, 1.15)	0.92 (0.79, 1.06)	0.94 (0.81, 1.09)	0.84 (0.72, 0.97)	0.01	
**Men**								
African-American	20,648 (2,518)	132,003	1.00 (0.89, 1.13)	0.95 (0.84, 1.07)	0.93 (0.82, 1.05)	0.85 (0.74, 0.97)	0.007	0.35
White or other race/ethnicity	10,540 (1,154)	59,528	0.92 (0.77, 1.09)	0.87 (0.72, 1.04)	0.96 (0.80, 1.15)	0.75 (0.62, 0.91)	0.01	
Household income < US$15,000/y	16,810 (2,542)	102,354	0.97 (0.86, 1.09)	0.91 (0.81, 1.03)	0.91 (0.81, 1.03)	0.79 (0.69, 0.91)	<0.001	0.98
Household income ≥ US$15,000/y	14,378 (1,130)	89,177	0.98 (0.80, 1.19)	0.91 (0.74, 1.10)	0.98 (0.81, 1.19)	0.83 (0.68, 1.02)	0.08	
**Women**								
African-American	29,786 (2,096)	196,778	1.00 (0.87, 1.15)	1.03 (0.90, 1.19)	0.95 (0.82, 1.10)	0.75 (0.64, 0.88)	<0.001	0.44
Whites or other race/ethnicity	16,598 (1,138)	96,435	1.02 (0.86, 1.22)	0.94 (0.78, 1.13)	0.87 (0.72, 1.06)	0.85 (0.70, 1.04)	0.04	
Household income < US$15,000/y	25,949 (2,376)	163,846	1.00 (0.89, 1.14)	1.02 (0.90, 1.16)	0.93 (0.82, 1.07)	0.75 (0.66, 0.87)	<0.001	0.82
Household income ≥ US$15,000/y	20,435 (858)	129,366	1.00 (0.80, 1.26)	0.93 (0.75, 1.17)	0.90 (0.72, 1.13)	0.84 (0.67, 1.06)	0.09	
**African-American**								
Household income < US$15,000/y	30,022 (3,407)	194,173	0.98 (0.89, 1.09)	0.97 (0.87, 1.08)	0.94 (0.84, 1.04)	0.76 (0.68, 0.86)	<0.001	0.63
Household income ≥ US$15,000/y	20,412 (1,207)	134,608	1.09 (0.89, 1.33)	1.05 (0.86, 1.28)	0.98 (0.81, 1.20)	0.92 (0.75, 1.12)	0.10	
**White or other race/ethnicity**								
Household income < US$15,000/y	12,737 (1,511)	72,027	1.00 (0.86, 1.16)	0.95 (0.81, 1.11)	0.91 (0.77, 1.07)	0.82 (0.69, 0.97)	0.02	0.50
Household income ≥ US$15,000/y	14,401 (781)	83,936	0.89 (0.71, 1.12)	0.78 (0.62, 0.98)	0.92 (0.73, 1.15)	0.75 (0.59, 0.95)	0.04	

Age was used as the underlying time scale, and wherever applicable, HRs were adjusted for race, enrollment source, education, income, marital status, medical insurance, cigarette smoking, BMI, physical activity, sitting time, total energy intake, menopausal status and hormone therapy in women, and baseline disease status.

No significant modifying effect of smoking and baseline disease status was found in the association between HEI-2010 score and total mortality (S2 Table; all *p*-values for interaction > 0.20). Similar results were found after excluding participants who died in the first 2 y of follow-up, participants recruited from the general population, or participants with uncertain cause of death (S3 Table). The association between HEI-2010 score and total mortality was essentially unchanged regardless of the approach used to treat missing data.

We identified 2,244 CVD deaths, 1,794 cancer deaths, and 2,550 deaths from other diseases (Table 3). HEI-2010 score was significantly associated with an approximately 20% lower risk comparing the extreme quintiles for cause-specific deaths (all *p*-values for trend < 0.05, except for cancer deaths in the white and other racial/ethnic groups). No significant heterogeneity was observed by race/ethnicity (all *p*-values for interaction > 0.50). The associations were similar when deaths other than from the cause of interest were censored or treated as competing events. In competing-risks analyses, the HRs (95% CIs) in the highest quintile of HEI-2010 were 0.82 (0.71–0.95) for cardiovascular deaths, 0.82 (0.70–0.96) for cancer deaths, and 0.78 (0.68–0.89) for other disease deaths (all *p*-values for trend < 0.04).

**Table 3:** Association of HEI-2010 score and risk of death from cardiovascular disease, cancer, or other diseases in the Southern Community Cohort Study, 2002–2011.

Population	Number of Deaths	Multivariate HR (95% CI) by Quintiles of HEI-2010 Score					*p* for Trend
		Quintile 1 (Low)	Quintile 2	Quintile 3	Quintile 4	Quintile 5 (High)	
**Total population**							
CVD deaths	2,244	1 (ref)	1.05 (0.92, 1.19)	1.00 (0.88, 1.15)	0.96 (0.84, 1.10)	0.81 (0.70, 0.94)	0.001
Cancer deaths	1,794	1 (ref)	0.86 (0.75, 1.00)	0.89 (0.77, 1.03)	0.94 (0.82, 1.09)	0.81 (0.69, 0.95)	0.047
Deaths from other diseases	2,550	1 (ref)	1.03 (0.91, 1.16)	0.97 (0.86, 1.09)	0.91 (0.80, 1.03)	0.77 (0.67, 0.88)	<0.001
**African-American**							
CVD deaths	1,540	1 (ref)	1.09 (0.92, 1.28)	1.02 (0.87, 1.20)	0.99 (0.84, 1.16)	0.85 (0.71, 1.01)	0.02
Cancer deaths	1,172	1 (ref)	0.86 (0.72, 1.03)	0.94 (0.79, 1.12)	0.91 (0.76, 1.09)	0.79 (0.64, 0.96)	0.05
Deaths from other diseases	1,663	1 (ref)	1.03 (0.89, 1.20)	1.00 (0.86, 1.16)	0.91 (0.78, 1.07)	0.78 (0.66, 0.92)	0.001
**White or other race/ethnicity**							
CVD deaths	704	1 (ref)	0.98 (0.78, 1.23)	0.98 (0.78, 1.23)	0.90 (0.71, 1.15)	0.73 (0.57, 0.95)	0.02
Cancer deaths	622	1 (ref)	0.86 (0.67, 1.09)	0.79 (0.61, 1.02)	1.01 (0.79, 1.29)	0.86 (0.66, 1.11)	0.49
Deaths from other diseases	887	1 (ref)	1.04 (0.86, 1.27)	0.92 (0.75, 1.13)	0.91 (0.73, 1.12)	0.78 (0.62, 0.99)	0.02

Age was used as the underlying time scale, and wherever applicable, HRs were adjusted for race, enrollment source, education, income, marital status, medical insurance, cigarette smoking, BMI, physical activity, sitting time, total energy intake, menopausal status and hormone therapy in women, and baseline disease status. Men and women were analyzed separately and combined using fixed-effect meta-analyses (all *p* for heterogeneity > 0.05). No significant interactions between HEI score and race were found; *p* for interaction = 0.69, 0.54, and 0.73 for CVD deaths, cancer deaths, and deaths from other diseases, respectively.

We evaluated the contribution of each component score in the HEI-2010 (Table 4). Four components—whole grains, dairy, seafood and plant proteins, and the ratio of unsaturated to saturated fatty acids—showed statistically significant, independent associations with lower disease mortality. The component score for fruit intake was not associated with total mortality; however, after further adjustment for the rest of the HEI components, scores for total and whole fruit intake showed unexpected adverse associations. Intakes of recommended food groups, distributions of component scores, and correlations between component scores and total score can be found in S4 and S5 Tables.

**Table 4:** Association of HEI-2010 component scores with total disease mortality in the Southern Community Cohort Study, 2002–2011.

HEI-2010 Component (Maximum Score)	Standard for Maximum Score^1^	Standard for Zero Score^1^	Model 1		Model 2	
			HR (95% CI) per 1-SD Increase^2^	*p*-Value	HR (95% CI) per 1-SD Increase^2^	*p*-Value
Total fruit (5)^3^	≥0.8 cup eq./1,000 kcal	No fruit	0.99 (0.96, 1.01)	0.30	1.04 (1.01, 1.07)	0.004
Whole fruit (5)^3^	≥0.4 cup eq./1,000 kcal	No whole fruit	0.99 (0.97, 1.02)	0.48	1.04 (1.01, 1.07)	0.003
Total vegetables (5)^4^	≥1.1 cup eq./1,000 kcal	No vegetables	0.97 (0.95, 1.00)	0.02	1.00 (0.94, 1.05)	0.93
Greens and beans (5)^4^	≥0.2 cup eq./1,000 kcal	No dark green vegetables, beans, or peas	0.96 (0.94, 0.99)	0.003	0.98 (0.96, 1.01)	0.21
Whole grains (10)	≥1.5 ounce eq./1,000 kcal	No whole grains	0.93 (0.90, 0.95)	<0.001	0.94 (0.92, 0.96)	<0.001
Dairy (10)	≥1.3 cup eq./1,000 kcal	No dairy	0.97 (0.94, 0.99)	0.01	0.97 (0.94, 0.99)	0.007
Total protein foods (5)^4^	≥2.5 ounce eq./1,000 kcal	No protein foods	0.98 (0.93, 1.03)	0.35	0.98 (0.93, 1.03)	0.38
Seafood and plant proteins (5)^4^	≥0.8 ounce eq./1,000 kcal	No seafood or plant proteins	0.93 (0.91, 0.95)	<0.001	0.95 (0.92, 0.97)	<0.001
(PUFAs + MUFAs)/SFAs (5)	>2.5	<1.2	0.96 (0.93, 0.98)	<0.001	0.97 (0.95, 0.99)	0.01
Refined grains (10)	≤1.8 ounce eq./1,000 kcal	≥4.3 ounce eq./1,000 kcal	0.97 (0.95, 0.99)	0.008	0.98 (0.96, 1.00)	0.11
Sodium (10)	≤1.1 g/1,000 kcal	≥2.0 g/1,000 kcal	1.00 (0.97, 1.02)	0.87	0.98 (0.96, 1.01)	0.19
Empty calories (20)^5^	≤19% of energy intake	≥50% of energy intake	0.96 (0.94, 0.99)	0.003	1.00 (0.97, 1.02)	0.82

^1^Intakes between the minimum and maximum levels were scored proportionately.

^2^Model 1 was adjusted for the same variables as in Table 2. Model 2 was further adjusted for a standardized score without the corresponding component. Men and women were analyzed separately and combined by fixed-effect meta-analyses for most components (all *p* for heterogeneity > 0.05), except that a random-effect model was used for total vegetables and total protein foods (*p* for heterogeneity = 0.03 and 0.02, respectively).

^3^100% fruit juice was included in total fruit, but not in whole fruit.

^4^Beans and peas were first counted as total protein foods and plant proteins; the excess amount was included in vegetables and beans.

^5^Calories from solid fats, alcohol, and added sugars; threshold for counting alcohol was >13 g/1,000 kcal.

eq., equivalent; MUFAs, monounsaturated fatty acids; PUFAs, polyunsaturated fatty acids; SFAs, saturated fatty acids.

The mean HEI-2005 score in the SCCS was 59.6 (SD: 11.1). It was strongly correlated with HEI-2010 score, with a Pearson correlation coefficient of 0.91. HEI-2005 score also showed inverse associations with total and cause-specific mortality, but with smaller magnitudes than HEI-2010 score in the total SCCS population or in any subgroup. The HRs (95% CIs) for total mortality comparing the extreme quintiles of HEI-2005 score were 0.86 (0.79–0.93) for all participants, 0.87 (0.78–0.96) for African-Americans, and 0.84 (0.76–0.93) for participants with a household income < US$15,000/y (all *p-*values for trend < 0.001). Results for total disease mortality by quintiles of HEI-2005 score can be found in S6 Table.

## Discussion

In this large, prospective cohort study of southeastern American adults, higher adherence to the DGA, as assessed by the HEI, was associated with 14%–23% lower mortality from all diseases, CVD, cancer, and other diseases. This association was found for all subgroups of participants in the SCCS, which is one of the largest studies of African-American and low-income populations in the United States. Four food groups, emphasized in the HEI-2010, showed significant inverse associations with total disease mortality in our study population, supporting the recommendations for increasing whole grains, dairy, seafood and plant proteins, and the ratio of unsaturated to saturated fatty acids in diets.

Among numerous nutrition findings, healthy dietary patterns and overall diet quality—rather than single nutrients or specific foods—have been consistently associated with lower morbidity and mortality from major chronic diseases [28,29]. In the SCCS, we evaluated diet quality via HEI, an a priori score based on the DGA, the most authoritative dietary recommendations for Americans [1]. Several large, well-characterized US cohorts, including the National Institutes of Health (NIH)–AARP (formerly known as the American Association of Retired Persons) Diet and Health Study, the Women’s Health Initiative observational study, the Nurses’ Health Study, and the Health Professionals Follow-Up Study, have reported that the highest quintile of HEI score was associated with 15%–25% decreases in total, CVD, and cancer mortality [12,30], as well as 10%–25% decreases in incident CVD, cancer, diabetes, or non-trauma deaths [5]. While those studies included Americans of predominantly European ancestry and middle-to-high income levels, our study provides novel and important evidence for African-American and low-income populations: the highest quintile of HEI-2010 score was associated with approximately 20% lower risks of total and disease-specific death, with similar and robust associations in stratified and sensitivity analyses. These consistent findings suggest that healthy dietary patterns, as described in the DGA, provide a universal benefit regardless of race/ethnicity, SES, and lifestyle choices. Among males, the associations between HEI score and mortality outcomes were, in general, linear, although most of the point estimates for HRs were statistically significant only in the highest quintile. Among females, however, the risk reduction was not seen until the fourth quintile of HEI score and, similar to males, was statistically significant only in the highest quintile, suggesting a possible threshold effect. It is possible that this seeming threshold effect could be due to chance alone, and additional research is needed.

Although evidence indicates that healthy eating could benefit the general US population, disparities exist by race/ethnicity and SES regarding diet quality and adherence to dietary guidelines. For example, the 20th–80th percentile of HEI-2010 score was 49–65 in the SCCS, lower than that observed in the NIH-AARP study (55–74) and the Women’s Health Initiative study (58–76) [12,30]. Within each study, as shown in the NHANES [17,31] and the present analysis, diet quality significantly improves with higher household income and educational attainment. Others have reported that African-Americans have lower diet quality scores than white and Hispanic individuals, but the differences greatly diminish when SES is similar or is controlled for [32]. Better diet quality was found for participants who were women, were older, had healthy lifestyle practices (such as nonsmoking and exercise), or had been diagnosed with some chronic diseases. After adjustment for these covariates, we did not find significant heterogeneity among races or income levels in the association of mortality with HEI score. To our knowledge, no previous study has evaluated the interaction between race/ethnicity and adherence to dietary guidelines in relation to disease mortality. In a study of a multi-ethnic, urban American population (55% Hispanic, 21% white, and 24% African-American), no significant racial differences were found in the association between a Mediterranean-style diet and risk of vascular death [33]. However, differential associations by race have been reported for dietary adherence scores and weight gain [3,20], insulin resistance [21], and incident diabetes [19], with inverse associations between scores and disease incidence seen in white individuals but null or positive associations in African-Americans. Racial differences may occur because of differences in income, education, access to healthy foods and exercise facilities, food and cooking preferences, sociopsychological factors, and biological mechanisms [14–16]. To date, most multi-ethnic studies have had relatively small sample sizes and have examined different health outcomes with various covariates. Our study, with its large sample size of African-American and white individuals who have comparable and generally low SES, its prospective design, and its careful adjustment of possible confounders, provides strong evidence that a DGA-accordant diet may reduce total and cause-specific mortality in low-income US adults including African-Americans.

Our study focused on dietary patterns that represent a combination of foods and their synergistic effects, but we also analyzed component scores separately to identify possible components that drive the association of HEI score with mortality. Each 1-SD increase in the total HEI score was associated with an 8% lower total mortality, which is stronger than the association for any single component score. This further supports the superiority of using a food-based total diet approach, as recognized by an increasing number of nutritional scientists [34–36] and endorsed by the US Academy of Nutrition and Dietetics [37]. Among HEI components, we observed statistically significant and independent inverse associations with disease mortality for recommendations on dairy, whole grains, seafood and plant proteins, and the ratio of unsaturated to saturated fatty acids. These last three components were newly added or particularly emphasized in HEI-2010 [10]. A number of epidemiological studies have shown that whole grain intake is associated with lower risk of type 2 diabetes [38], CVD [39], colorectal cancer [40], and related mortality [5,12,41,42]. Whole grains, in contrast to refined grains, are rich sources of fiber, minerals, vitamins, and phytochemicals, and may improve a wide range of metabolic disorders, alleviate oxidative stress and inflammation, and nourish gut microbiota [43]. Notably, only 3.4% of men and 5.4% of women in the SCCS met the recommendation of consuming ≥3 ounce equivalents of whole grains per day (for a 2,000 kcal diet). In contrast, approximately 85% of participants consumed more refined grains than the recommended 3.6 ounce equivalents. These data indicate that the quality of grain intake in the SCCS population is far from optimal, which agrees with NHANES data showing that the score for whole grain intake was among the lowest [44], particularly in the low-income population [45]. Strong evidence also supports the benefits of consuming foods rich in healthy protein and fat [28,46–48], including seafood, legumes, nuts, and seeds, especially when they substitute for less healthy protein sources, e.g., red meat and processed meat [49]. These healthy protein foods have been recommended in many eating plans, including the DGA, the Dietary Approaches to Stop Hypertension [50], the Harvard Healthy Eating Pyramid [5], and the Mediterranean diet [48]. To our surprise, component scores for total and whole fruit intake were positively associated with total mortality in SCCS participants when the rest of HEI score was adjusted. The adverse association was mainly observed in deaths other than from CVD and cancer and remained statistically significant after excluding the first 2 y of follow-up. In the NIH-AARP study, fruit components also showed positive associations with liver cancer and liver disease mortality [13], in contrast to the inverse associations with all-cause, CVD, and total cancer mortality [12]. The reasons for this observation are unclear. Nevertheless, we believe that emphasis should be placed on dietary patterns rather than single food groups, and the results of individual component scores should be interpreted cautiously because of the possibility of false-positive findings due to collinearity and multiple comparisons.

As with any observational study, dietary assessment in our study is prone to participants’ reporting errors. Because of the prospective design, these errors are likely to be non-differential between those who subsequently died during follow-up and those who survived to the end of the study. We used energy-adjusted intakes to reduce possible influence of over- or underreporting in our results. Participants with diseases at baseline had higher HEI scores than healthy participants, suggesting that a disease diagnosis might prompt lifestyle changes. To address this possible bias, we excluded the mortality outcomes identified in the first 2 y of follow-up, adjusted for common diseases, and performed stratified analyses by disease status. All these analyses yielded similar results, suggesting that recent dietary changes among participants with comorbidity may not appreciably affect our results. Furthermore, this influence, if any, would bias the results towards null, suggesting that the true association between HEI score and disease mortality could be stronger than what we observed in this study. Diet quality is associated with health-related behaviors and social and psychological factors. Although we carefully controlled for potential confounders, some residual confounding is still possible. The present study was conducted primarily in a low-income population. The generalizability of these findings to African-Americans of other SES should be evaluated.

In summary, in this large prospective cohort study of African-American and white individuals living in the southeastern US, higher adherence to the DGA was associated with lower mortality from all diseases combined, as well as from CVD, cancer, and other diseases. This is the first study to our knowledge reporting this association in a low-income population that largely comprises African-Americans, providing direct evidence for disease prevention through dietary modification in this underserved population. Our study also suggests that dietary factors may explain some of the disparity in disease mortality by race/ethnicity and SES.

## Supporting Information

S1 Table Age-adjusted Healthy Eating Index scores by sex, race, and income in the Southern Community Cohort Study, 2002–2009. (DOCX)

S2 Table Association of HEI-2010 score and total disease mortality by baseline cigarette smoking and disease status in the Southern Community Cohort Study, 2002–2011. (DOCX)

S3 Table Sensitivity and imputation analyses for the association between HEI-2010 score and total disease mortality in the Southern Community Cohort Study, 2002–2011. (DOCX)

S4 Table Baseline dietary intakes by sex-specific quintiles of HEI-2010 scores in the Southern Community Cohort Study, 2002–2009. (DOCX)

S5 Table Distribution of HEI-2010 component scores in the Southern Community Cohort Study, 2002–2009. (DOCX)

S6 Table Association of HEI-2005 score and total disease mortality by sex, race, and income in the Southern Community Cohort Study, 2002–2011. (DOCX)

S1 Text STROBE checklist. (DOC)

S2 Text Analysis plan. (PDF)

## Abbreviations

BMI: body mass index
CHC: community health center
COPD: chronic obstructive pulmonary disease
CVD: cardiovascular disease
DGA: Dietary Guidelines for Americans
FFQ: food frequency questionnaire
HEI: Healthy Eating Index
HEI-2005: Healthy Eating Index, 2005 edition
HEI-2010: Healthy Eating Index, 2010 edition
HR: hazard ratio
ICD-10: International Classification of Diseases, 10th Revision
NDI: National Death Index
NHANES: National Health and Nutrition Examination Survey
NIH: National Institutes of Health
SCCS: Southern Community Cohort Study
SD: standard deviation
SES: socioeconomic status
SSA: Social Security Administration
USDA: US Department of Agriculture
