# Supplementary material for: Healthy Eating and Risks of Total and Cause-Specific Death among Low-Income Populations of African-Americans and Other Adults in the Southeastern United States: A Prospective Cohort Study
Source: PLoS Med. 2015 May 26;12(5):e1001830. doi: 10.1371/journal.pmed.1001830 (PMC4444091; doi:10.1371/journal.pmed.1001830)
Supplement: S1 Table — (DOCX) [file pmed.1001830.s001.docx]

**S1 Table.** Age-adjusted Healthy Eating Index (HEI) by sex, race, and income in the Southern Community Cohort Study, 2002-2009

| **Populations** | | **No. of participants** | **HEI-2005** | | | **HEI-2010** | | |
| --- | --- | --- | --- | --- | --- | --- | --- | --- |
|  |  |  | **Mean ± SD** | **Mean difference** | **P for difference** | **Mean ± SD** | **Mean difference** | **P for difference** |
| Total | |  |  |  |  |  |  |  |
|  | Men | 31,188 | 56.8 ± 10.4 | 4.7 | <0.001 | 55.2 ± 11.5 | 4.2 | <0.001 |
|  | Women | 46,384 | 61.5 ± 10.4 |  |  | 59.4 ± 11.5 |  |  |
| Men | |  |  |  |  |  |  |  |
|  | African Americans | 20,648 | 56.7 ± 10.6 | -0.3^1^ | 0.02 | 55.3 ± 11.3 | -0.6^1^ | <0.001 |
|  | Whites | 9,406 | 56.4 ± 10.7 |  |  | 54.7 ± 11.4 |  |  |
|  | Other racial/ethnic groups | 1,134 | 59.0 ± 11.3 | 2.3^1^ | <0.001 | 57.5 ± 12.1 | 2.2^1^ | <0.001 |
| Women | |  |  |  |  |  |  |  |
|  | African Americans | 29,786 | 61.8 ± 10.4 | -1.1^1^ | <0.001 | 60.0 ± 11.7 | -1.6^1^ | <0.001 |
|  | Whites | 14,648 | 60.7 ± 10.4 |  |  | 58.3 ± 11.7 |  |  |
|  | Other racial/ethnic groups | 1,950 | 62.9 ± 11.1 | 1.1^1^ | <0.001 | 61.1± 12.3 | 1.1^1^ | <0.001 |
| Men | |  |  |  |  |  |  |  |
|  | Household income < $15,000/year | 16,810 | 55.2 ± 10.5 | 3.1 | <0.001 | 53.5 ± 11.2 | 3.5 | <0.001 |
|  | Household income ≥ $15,000/year | 14,378 | 58.3 ± 10.5 |  |  | 57.0 ± 11.2 |  |  |
| Women | |  |  |  |  |  |  |  |
|  | Household income < $15,000/year | 25,949 | 60.3 ± 10.3 | 2.7 | <.001 | 58.0 ± 11.6 | 3.3 | <.001 |
|  | Household income ≥ $15,000/year | 20,435 | 63.0 ± 10.3 |  |  | 61.3 ± 11.6 |  |  |

^1^Compared with African Americans. Other racial/ethnic groups included Hispanic/Latino, American Indian or Alaska Native, Asian or Pacific Islander, mixed race, and others.
