# Supplementary material for: Healthy Eating and Risks of Total and Cause-Specific Death among Low-Income Populations of African-Americans and Other Adults in the Southeastern United States: A Prospective Cohort Study
Source: PLoS Med. 2015 May 26;12(5):e1001830. doi: 10.1371/journal.pmed.1001830 (PMC4444091; doi:10.1371/journal.pmed.1001830)
Supplement: S2 Table — (DOCX) [file pmed.1001830.s002.docx]

**S2 Table.** Association of Healthy Eating Index (HEI)-2010 and total disease mortality by baseline cigarette smoking and disease status in the Southern Community Cohort Study, 2002-2011

| **Populations** | | **No. of participants (deaths)** | **Multivariate HR (95% CI) by HEI-2010** | | |
| --- | --- | --- | --- | --- | --- |
|  |  |  | **Quintile 5 vs. Quintile 1** | **P for trend** | **P for interaction** |
| Men^1^ | |  |  |  |  |
|  | Non-smokers | 7,300 (607) | 0.94 (0.71, 1.23) | 0.42 | 0.54 |
|  | Former smokers | 7,783 (945) | 0.76 (0.61, 0.93) | 0.004 |  |
|  | Current smokers | 16,105 (2,120) | 0.81 (0.70, 0.95) | 0.01 |  |
| Women^1^ | |  |  |  |  |
|  | Non-smokers | 21,088 (1,085) | 0.67 (0.55, 0.83) | <0.001 | 0.42 |
|  | Former smokers | 9,922 (748) | 0.87 (0.67, 1.13) | 0.13 |  |
|  | Current smokers | 15,374 (1,401) | 0.86 (0.70, 1.05) | 0.12 |  |
| Men^2^ | |  |  |  |  |
|  | Severe diseases | 6,288 (1,296) | 0.82 (0.69, 0.99) | 0.02 | 0.89 |
|  | Other diseases | 14,514 (1,690) | 0.82 (0.70, 0.96) | 0.01 |  |
|  | No diseases | 10,386 (686) | 0.87 (0.66, 1.15) | 0.42 |  |
| Women^2^ | |  |  |  |  |
|  | Severe diseases | 9,073 (1,137) | 0.93 (0.75, 1.15) | 0.23 | 0.22 |
|  | Other diseases | 26,277 (1,714) | 0.78 (0.66, 0.92) | 0.004 |  |
|  | No diseases | 11,034 (383) | 0.71 (0.48, 1.05) | 0.09 |  |

^1^Age as the underlying timescale and wherever applicable, adjusted for race, enrollment source, education, income, marital status, medical insurance, body mass index, physical activity, sitting time, total energy intake, and menopausal status and hormone therapy in women, and baseline diseases.

^2^Age as the underlying timescale and wherever applicable, adjusted for race, enrollment source, education, income, marital status, medical insurance, cigarette smoking, body mass index, physical activity, sitting time, total energy intake, and menopausal status and hormone therapy in women. Severe diseases were defined as having cardiovascular disease, cancer, or HIV/AIDS. Other diseases were defined as having hypertension, diabetes, hypercholesterolemia, or COPD, without severe diseases listed above. No diseases were defined as having no self-reported diseases listed above.
