# Supplementary material for: Healthy Eating and Risks of Total and Cause-Specific Death among Low-Income Populations of African-Americans and Other Adults in the Southeastern United States: A Prospective Cohort Study
Source: PLoS Med. 2015 May 26;12(5):e1001830. doi: 10.1371/journal.pmed.1001830 (PMC4444091; doi:10.1371/journal.pmed.1001830)
Supplement: S3 Table — (DOCX) [file pmed.1001830.s003.docx]

**S3 Table.** Sensitivity and imputation analyses for the association between Healthy Eating Index (HEI)-2010 and total disease mortality in the Southern Community Cohort Study, 2002-2011

| **Populations** | | **No. of participants (deaths)** | **Multivariate HR (95% CI) by HEI-2010** | |
| --- | --- | --- | --- | --- |
|  |  |  | **Quintile 5 vs. Quintile 1** | **P for trend** |
| Excluding participants died in the first 2 years of follow-up | | | | |
|  | Men | 30,239 (2,826) | 0.78 (0.69, 0.88) | <0.001 |
|  | Women | 45,611 (2,520) | 0.78 (0.68, 0.89) | <0.001 |
| Excluding participants not recruited from CHCs | | | | |
|  | Men | 26,135 (3,304) | 0.83 (0.74, 0.93) | 0.002 |
|  | Women | 40,846 (2,976) | 0.79 (0.69, 0.89) | <0.001 |
| Excluding participants with uncertain causes of death | | | | |
|  | Men | 31,036 (3,520) | 0.81 (0.72, 0.90) | <0.001 |
|  | Women | 46,218 (3,068) | 0.78 (0.68, 0.88) | <0.001 |
| Excluding participants with any missing information | | | | |
|  | Men | 28,851 (3,400) | 0.80 (0.72, 0.90) | <0.001 |
|  | Women | 42,805 (2,961) | 0.78 (0.68, 0.88) | <0.001 |
| Conducting multiple imputation for unreliable dietary data and other missing variables | | | | |
|  | Men | 33,944 (3,983) | 0.80 (0.76, 0.84) | <0.001 |
|  | Women | 49,967 (3,518) | 0.79 (0.74, 0.83) | <0.001 |

^1^Age as the underlying timescale and wherever applicable, adjusted for race, enrollment source, education, income, marital status, medical insurance, cigarette smoking, body mass index, physical activity, sitting time, total energy intake, and menopausal status and hormone therapy in women, and baseline diseases.
