# Supplementary material for: Healthy Eating and Risks of Total and Cause-Specific Death among Low-Income Populations of African-Americans and Other Adults in the Southeastern United States: A Prospective Cohort Study
Source: PLoS Med. 2015 May 26;12(5):e1001830. doi: 10.1371/journal.pmed.1001830 (PMC4444091; doi:10.1371/journal.pmed.1001830)
Supplement: S4 Table — (DOCX) [file pmed.1001830.s004.docx]

**S4 Table.** Baseline dietary intakes by sex-specific quintiles of Healthy Eating Index-2010 in the Southern Community Cohort Study, 2002-2009^1^

| **Dietary intakes, per 1,000 kcal** | | **Men (n = 31,188)** | | | **Women (n = 46,384)** | | |
| --- | --- | --- | --- | --- | --- | --- | --- |
|  |  | **Q1** | **Q3** | **Q5** | **Q1** | **Q3** | **Q5** |
|  | Total energy, kcal/day | 3089 | 3185 | 2709 | 2328 | 2295 | 1899 |
|  | Total fruit, cup equivalent | 0.2 | 0.6 | 1.3 | 0.3 | 0.8 | 1.6 |
|  | Whole fruit, cup equivalent | 0.1 | 0.3 | 0.8 | 0.2 | 0.5 | 1.1 |
|  | Total vegetables, cup equivalent | 0.6 | 0.9 | 1.2 | 0.8 | 1.1 | 1.5 |
|  | Whole grains, oz equivalent | 0.2 | 0.4 | 0.8 | 0.3 | 0.5 | 1.0 |
|  | Refine grains, oz equivalent | 2.9 | 2.7 | 2.2 | 3.0 | 2.6 | 1.9 |
|  | Total dairy, cup equivalent | 0.4 | 0.4 | 0.5 | 0.5 | 0.5 | 0.6 |
|  | Total protein foods, oz equivalent | 2.9 | 3.2 | 3.1 | 2.9 | 3.1 | 3.1 |
|  | Seafood and plant protein, oz equivalent | 0.6 | 0.9 | 1.4 | 0.6 | 0.9 | 1.4 |
|  | Red and processed meat, oz equivalent | 1.5 | 1.4 | 0.9 | 1.4 | 1.2 | 0.7 |
|  | (MUFA+PUFA) / SFA | 1.9 | 2.0 | 2.3 | 1.9 | 2.1 | 2.3 |
|  | Sodium, mg/day | 1696 | 1761 | 1653 | 1710 | 1743 | 1647 |
|  | Alcohol, g/day | 13.1 | 7.0 | 5.4 | 4.4 | 2.6 | 2.1 |
|  | Calories from SoFAAS, % of energy | 41.2 | 33.1 | 24.9 | 40.6 | 32.0 | 23.1 |

Abbreviations: MUFA = monounsaturated fatty acids; PUFA = polyunsaturated fatty acids; SFA = saturated fatty acids; SoFAAS = solid fats, alcohol, and added sugars.

^1^ Age- and energy-adjusted mean.
