# Supplementary material for: Healthy Eating and Risks of Total and Cause-Specific Death among Low-Income Populations of African-Americans and Other Adults in the Southeastern United States: A Prospective Cohort Study
Source: PLoS Med. 2015 May 26;12(5):e1001830. doi: 10.1371/journal.pmed.1001830 (PMC4444091; doi:10.1371/journal.pmed.1001830)
Supplement: S5 Table — (DOCX) [file pmed.1001830.s005.docx]

**S5 Table.** Distribution of Healthy Eating Index-2010 component scores in the Southern Community Cohort Study, 2002-2009

| **Component (maximum score)** | **r^1^** | **Men** | | **Women** | |
| --- | --- | --- | --- | --- | --- |
|  |  | **Median score (IQR)** | **% of maximum** | **Median score (IQR)** | **% of maximum** |
| Total fruit (5) | 0.64 | 2.9 (1.3, 5.0) | 28.6 | 4.2 (1.9, 5.0) | 42.7 |
| Whole fruit (5) | 0.63 | 2.9 (1.1, 5.0) | 32.7 | 4.8 (1.9, 5.0) | 48.4 |
| Total vegetables (5) | 0.44 | 3.8 (2.7, 5.0) | 26.8 | 4.6 (3.3, 5.0) | 42.6 |
| Greens and beans (5) | 0.43 | 4.0 (2.0, 5.0) | 39.9 | 5.0 (2.6, 5.0) | 49.8 |
| Whole grains (10) | 0.54 | 2.3 (0.9, 4.3) | 3.4 | 2.9 (1.2, 5.3) | 5.4 |
| Dairy (10) | 0.19 | 2.9 (1.7, 4.5) | 1.8 | 3.4 (2.1, 5.3) | 3.6 |
| Total protein foods (5) | 0.13 | 5.0 (5.0, 5.0) | 77.5 | 5.0 (4.9, 5.0) | 72.8 |
| Seafood and plant proteins (5) | 0.42 | 4.8 (3.0, 5.0) | 48.0 | 4.9 (3.0, 5.0) | 48.5 |
| (MUFA+PUFA)/SFA (10) | 0.39 | 6.4 (5.0, 7.8) | 7.5 | 6.7 (5.2, 8.4) | 11.1 |
| Refined grains (10) | 0.42 | 6.7 (4.8, 8.8) | 14.2 | 7.1 (5.1, 9.2) | 17.9 |
| Sodium (10) | 0.05 | 2.9 (0.7, 5.2) | 4.6 | 3.1 (1.0, 5.3) | 2.8 |
| Calories from SoFAAS (20) | 0.74 | 11.3 (8.1, 14.1) | 2.5 | 12.0 (8.6, 15.2) | 4.6 |

Abbreviations: IQR = interquartile range; MUFA = monounsaturated fatty acids; PUFA = polyunsaturated fatty acids; SFA = saturated fatty acids; SoFAAS = solid fats, alcohol, and added sugars.

^1^ Pearson correlation coefficients between component scores and the total HEI-2010 score, with adjustment for age, sex, and race.
