# Supplementary material for: Healthy Eating and Risks of Total and Cause-Specific Death among Low-Income Populations of African-Americans and Other Adults in the Southeastern United States: A Prospective Cohort Study
Source: PLoS Med. 2015 May 26;12(5):e1001830. doi: 10.1371/journal.pmed.1001830 (PMC4444091; doi:10.1371/journal.pmed.1001830)
Supplement: S6 Table — (DOCX) [file pmed.1001830.s006.docx]

**S6 Table.** Association of Healthy Eating Index (HEI)-2005 and total disease mortality by sex, race, and income in the Southern Community Cohort Study, 2002-2011^1^

| **Populations** | **No. of participants (deaths)** | **Multivariate HR (95% CI) by Quintiles of HEI-2005, Q1 = referent** | | | | ***P* for trend** | ***P* for interaction** |
| --- | --- | --- | --- | --- | --- | --- | --- |
|  |  | **Q2** | **Q3** | **Q4** | **Q5 (high)** |  |  |
| Total population | 77,572 (6,906) | 1.00 (0.93, 1.08) | 0.97 (0.90, 1.05) | 0.91 (0.84, 0.98) | 0.86 (0.79, 0.93) | <0.001 |  |
| Men | 31,188 (3,672) | 0.97 (0.88, 1.07) | 0.94 (0.85, 1.04) | 0.86 (0.77, 0.95) | 0.86 (0.77, 0.96) | <0.001 | 0.70 |
| Women | 46,384 (3,234) | 1.04 (0.93, 1.16) | 1.02 (0.92, 1.14) | 0.97 (0.86, 1.08) | 0.87 (0.77, 0.98) | 0.01 |  |
| African Americans | 50,434 (4,614) | 1.02 (0.93, 1.11) | 1.00 (0.91, 1.10) | 0.88 (0.80, 0.97) | 0.87 (0.78, 0.96) | <0.001 | 0.27 |
| Whites and other racial/ethnic groups | 27,138 (2,292) | 0.98 (0.86, 1.11) | 0.92 0.81, 1.05) | 0.96 (0.84, 1.10) | 0.85 (0.74, 0.98) | 0.04 |  |
| Household income < $15,000/year | 42,759 (4,918) | 1.00 (0.92, 1.09) | 0.98 (0.90, 1.07) | 0.92 (0.84, 1.00) | 0.84 (0.76, 0.93) | <0.001 | 0.90 |
| Household income ≥ $15,000/year | 34,813 (1,988) | 1.00 (0.86, 1.17) | 0.96 (0.83, 1.12) | 0.87 (0.75, 1.02) | 0.90 (0.77, 1.05) | 0.06 |  |

^1^Age as the underlying timescale and wherever applicable, adjusted for race, enrollment source, education, income, marital status, medical insurance, cigarette smoking, body mass index, physical activity, sitting time, total energy intake, and menopausal status and hormone therapy in women, and baseline diseases.
