# Supplementary material for: Healthy Eating and Risks of Total and Cause-Specific Death among Low-Income Populations of African-Americans and Other Adults in the Southeastern United States: A Prospective Cohort Study
Source: PLoS Med. 2015 May 26;12(5):e1001830. doi: 10.1371/journal.pmed.1001830 (PMC4444091; doi:10.1371/journal.pmed.1001830)
Supplement: S2 Text — (PDF) [file pmed.1001830.s008.pdf]

DATA ACCESS PROPOSAL FORM

INSTRUCTIONS: Each section must be completed. If a section is not applicable to your submission, state so ("Not applicable") and explain exactly why the section is not applicable. The suggested maximum length is 6 pages (not including variables/references).

**1. Proposal Title:**
Diet quality, socioeconomic status, and risk of morbidity and mortality from major diseases
**2. Point(s) of contact:**
Applicant name: Danxia Yu and Wei Zheng<br>Title: Danxia Yu: Postdoctoral Research Fellow; Wei Zheng: Professor and Director<br>e-Mail: danxia.yu@vanderbilt.edu; wei.zheng@vanderbilt.edu<br>Organization: Vanderbilt Epidemiology Center
**3. Investigators/personnel involved in the analysis of the data, the interpretation of data results, and/or the writing of the resulting scientific manuscript(s). One of the listed investigators must be an SCCS Investigator.** *(Include qualifications of the person(s) performing the statistical analysis)*
Lead investigator: Danxia Yu and Wei Zheng<br>SCCS investigator(s): Wei Zheng, William Blot, and Margaret K. Hargreaves<br>Other investigators:<br>Data analysis personnel: Danxia Yu
**4. Purpose of Request** *(check all that apply)*:
☐ Abstract ☒ Manuscript ☐ Grant
**5. Plan for funding** *(include grant number, date due to funding institution, and project period if applicable)*:
SCCS and Ingram Professorship funds
**6. Timeline for work completion:**
12 months
**7. Proposal abstract** *(100 words or less)*:
A high-quality diet, assessed by conformance to healthy diet recommendations, has been associated with reduced morbidity and mortality from major chronic diseases, including cardiovascular diseases, diabetes, and some types of cancer. These associations, however, have not been adequately investigated in African Americans and low-income populations who tend to consume more energy-dense but nutrient-poor foods than whites and higher-income populations. We propose to use SCCS data to evaluate the associations of overall diet quality using multiple diet quality scores with total and cause-specific mortality and risk of major chronic diseases, and further explore the influence of sociodemographics and major disease risk factors on the diet-disease association.
**8. Keywords** *(5-7)*:
diet quality, sociodemographic characteristics, mortality, cancer, cardiovascular disease, diabetes
**9. Background (scientific justification for the proposed analysis):**
Analysis of overall diet quality focuses on the entire diet rather than a single food or nutrient, and thus it may help to detect possible synergistic effects of foods and nutrients and provide more direct information for developing dietary guidelines and recommendations. Diet quality can be measured by assessing conformance to recommended healthy diets or by data-driven pattern analyses [1]. Several studies, including our own study in Chinese adults [2], have suggested that better diet quality may be associated with decreased morbidity and mortality from multiple chronic

diseases [3]. For example, the Healthy Eating Index-2005 (HEI-2005), a measure of adherence to 2005 Dietary Guidelines for Americans [4], has been shown to be associated with 16% lower risk of major chronic disease, including cardiovascular disease (CVD), diabetes and cancer [5]. Another well-known diet quality score, the Alternative Healthy Eating Index (AHEI) was designed by Harvard researchers and consists of foods and nutrients which have been shown to have health benefits [6]. In the Nurses' Health Study and the Health Professionals Follow-up Study, the highest quintile of AHEI was associated with 20% lower risk of chronic diseases, with a stronger association observed for reduced risk of CVD and diabetes than for cancer [5]. A higher AHEI has also been associated with reduced risk of heart failure [7], colorectal cancer [8], ER-negative breast cancer [9], and total and CVD-specific mortality [10] in other U.S. populations. In addition to HEI-2005 and AHEI, several other diet quality scores have been proposed, such as the Dietary Approaches to Stop Hypertension (DASH) score and Mediterranean Diet Score [3]. However, most of these scores were created and investigated primarily in non-Hispanic whites or well-educated health professionals. Little is known about their utilities in predicting health outcomes in non-whites and low-income populations.

Certain sociodemographic characteristics, such as age, sex, race/ethnicity, and socioeconomic status (SES), have been shown to influence food choices and diet quality [11]. Individuals with a high SES tend to consume a more healthy diet consisting of whole grains, fresh fruits and vegetables, lean meats, fish, and low-fat dairy products compared with those with a low SES [11;12]. Disparities also exist across races/ethnicities, with non-Hispanic whites generally having a higher diet quality score than non-Hispanic blacks. In the 2003-04 National Health and Nutrition Examination Survey, the mean HEI-2005 was 53 vs. 56 for non-Hispanic blacks vs. whites, and 55 vs. 58 for low-income vs. high-income adults [12]. In comparison, the mean HEI-2005 was 62 in the Nurses' Health study and the Health Professionals Follow-up Study [5]. In a study with multi-ethnic populations, Wang et al. found that the black-white difference in HEI-2005 was reduced by 30% after adjustment for SES; however, a large portion of racial difference in HEI remained unexplained [13]. Over the past few decades, prices of healthy foods have risen more rapidly than unhealthy foods. It is possible that disparity in diet quality has widened and the gap will continue to increase in the future [14]. Thus, it is crucial to understand sociodemographic determinants of diet quality, especially among low-SES individuals and minorities in order to develop targeted interventions to reduce health disparities.

Additional unresolved questions are how sociodemographic characteristics, existing medical conditions, cigarette smoking, obesity and other disease risk factors modify the association between diet quality and disease risk. Universal dietary recommendations could lead to different dietary patterns. For example, recommendations for meat intake in the Dietary Guidelines for Americans do not distinguish red meat from white meat. It was reported that good adherence to national dietary guidelines was associated with less weight gain in young white adults, but increased weight gain in blacks, particularly in obese blacks [15]. Several recent studies also suggested possible modifying effects of smoking habits and obesity on the associations of diet quality and certain health outcomes [16;17]. The SCCS, with its unique study population, detailed dietary assessments, large sample size, and prospective study design, provides an excellent opportunity to address the influence of diet quality and its interactions with other risk factors in the etiology of major chronic diseases.

10. Hypothesis(es) to be tested:

We aim to:
1) Create diet quality scores based on commonly used dietary guidelines, including HEI-2005, AHEI, and DASH, and evaluate their associations with race/ethnicity, SES, smoking, obesity, and other major disease risk factors.
2) Examine associations of diet quality scores with total and cause-specific (cancer, CVD, other) mortality. In secondary analyses, we will also examine associations with risk of incident cancer (all types combined and lung, breast, prostate, and colorectal cancers) and self-reported major chronic diseases, including myocardial infarction, stroke, and diabetes.
3) Investigate potential modifying effects of selected sociodemographic characteristics, existing medical conditions, and major disease risk factors on diet quality-disease association.

DATA ACCESS PROPOSAL FORM

INSTRUCTIONS: Each section must be completed. If a section is not applicable to your submission, state so ("Not applicable") and explain exactly why the section is not applicable. The suggested maximum length is 6 pages (not including variables/references).

11. Number of participants involved and details of sample selection:
For mortality and cancer incidence analyses, we will include all SCCS participants who completed the baseline FFQ. For myocardial infarction, stroke and diabetes analyses, we will include all SCCS participants who completed follow-up one and/or two questionnaires.

12. Detailed analytic/statistical plan *(four paragraphs maximum; include power calculations)*:

We recently completed a project to investigate the association of diet quality with total and cause-specific mortality using data from the Shanghai Men's and Women's Health Studies and will use a similar approach to analyze SCCS data in this proposed project.

For Aim 1, we will calculate diet quality scores based on national and other prevailing dietary guidelines. Since recommendations were generally made according to daily energy requirements, dietary intakes will be adjusted for total energy (density method) and standardized to 2,000 kcal before analysis. For example, HEI-2005 contains 9 "adequacy" components: total fruit (including 100% juice), whole fruit, total vegetables, dark-green and orange vegetables and legumes, total grains, whole grains, milk (including soy beverages), meat and beans, and healthy oils. For this set of dietary components, a maximum 10 points will be assigned to each of these components for meeting recommended intakes. A minimum score, 0 points, will be assigned for no consumption. Intermediate intakes between minimum and maximum levels will be scored proportionately. Another set of dietary components will be scored based on a moderation criterion that higher consumption gets lower score, including saturated fat, sodium, and calories from solid fat, alcohol, and added sugar. All component scores will be summed to obtain a total HEI-2005 score, ranging from 0 to 100 (lowest to highest adherence)4. Dietary recommendations similarly emphasize consumption of whole grains, vegetables, fruits, nuts, and legumes. However, recommendations on dairy products, meats, sodium, and fat may be different. Thereby, calculations of various diet quality scores enable us to examine how robust findings are across indices and to compare our results with other studies. We will conduct cross-sectional analyses of these diet quality scores in relation to sex, race/ethnicity, SES, smoking, obesity, and other major known risk factors for chronic diseases. Major analyses will be aimed at evaluating disparities of diet quality by race and SES and how these disparities may be explained by other risk factors. For Aim 2, we will conduct analyses by sex and race. We will use Cox proportional hazard regression with age as the underlying time metric to examine associations between diet quality scores and risk of death (primary analyses). We will examine associations between diet quality scores and incident cancer and self-reported myocardial infarction, stroke, and diabetes (secondary analyses). Potential confounders will be adjusted when appropriate, such as education, household income, marital status, smoking, alcohol consumption, physical activity, use of multivitamin supplements, hormone replacement therapy (women only), body mass index, total energy intake, type of health insurance, and prior history of hypertension, diabetes, hypercholesterolemia, CVD, or cancer. We will test linear trends by treating the median value of each quintile as a continuous variable. We will also perform restricted cubic spline analyses to assess possible non-linear associations. Sensitivity analyses will be conducted by excluding the first year of follow-up or stratifying by baseline comorbidity. Additional analyses will be conducted to examine whether the association is driven by a specific dietary component by including all standardized component scores in one model or one component score plus standardized total score without corresponding component. For Aim 3, we will identify potential modifiers on the association between diet quality scores and risk of diseases and death. Maximum likelihood ratio tests with and without the interaction terms will be used to assess possible interactions under multiplicative models.

Study power analyses were based on cohort follow-up through the end of 2011 when nearly 8,000 deaths had been identified in the cohort. Provided below are the smallest detectable RRs by race and exposure prevalence for the primary analysis proposed for this study: associations of diet quality scores with total and cause-specific mortality. Exposure prevalence rates were selected to reflect categorical analyses by quintile (20%), quartile (25%), tertile (33%), or median (50%). Since most previous studies reported more than 30% elevated risk of deaths associated with poor diet quality scores, we predict that our statistical power should be adequate in our primary analyses.

Smallest detectable RRs (power=0.8, alpha=0.05)

	White (N=30447)			Black (N=55578)		
	Total	Cancer	CVD	Total	Cancer	CVD
Exp	N=2415	N=595	N=636	N=5537	N=1353	N=1559
20%	1.15	1.31	1.30	1.10	1.20	1.18
25%	1.14	1.29	1.28	1.09	1.18	1.17
33%	1.12	1.26	1.26	1.08	1.17	1.16
50%	1.12	1.26	1.25	1.08	1.16	1.15

To date, approximately 4500 incident cancer cases have been identified in the SCCS, including 623 cases of breast cancer, 617 cases of prostate cancer, 916 cases of lung cancer, and 439 cases of colorectal cancer. Statistical power for analyzing the association of diet quality scores with total and site-specific cancers should be adequate based on the power estimates provided in the table presented above. Statistical power for race-specific analyses could be low for some cancer sites. Nevertheless, for a site with 300 cases, we should be able to identify an association with a RR as low as 1.39 associated with the lowest tertile diet quality score with 80% statistical power (type I error = 0.05). Statistical power for studying incident type 2 diabetes should be high as approximately 2.5% of cohort members develop this disease annually. With an annual rate of approximately 0.6%, we have a relatively low statistical power to study incident CVD. However, with an extended follow-up of this cohort, the statistical power will be increased significantly in the coming years.

13. Listing of exact variable names (from the current SCCS Codebooks) requested in the dataset.
If a derived variable is requested (e.g., "incident COPD"), specify the data source(s), codes, and/or algorithm required *(see form instructions for an example of a sufficient request).*

We will work closely with staff of the SCCS coordinating center to identify appropriate variables for these analyses. Provided below is a tentative list of study variables.

Baseline codebook: Enrollment_Age, Sex, RaceAnalysis, Education, HHIncome, MaritalStatus, BMI, BMICat, SmokingStatus, AgeStartSmoking, AgeQuitSmoking, CigsPerDay, PackYears, Hypertension, Hypertension_Med, Diabetes, Diabetes_Med, MI_Bypass, HyperChol, HyperChol_Med, Stroke_TIA, Cancer, HRT_Ever, InsuranceCoverage, Employed, AlcoholPerDay, Dieting, Overeating, UnplannedSnacking, StressEating, RestaurantEating, MultiVit, SleepAverage_hrs, LtWorkAvgMetHr, ModActivityMetHr, VigActivityMetHr

All baseline dietary variables: from MeatPerDay to Sugar. And nutrient variables: FFQ_KCAL, FFQ_CARB, FFQ_PROT, FFQ_TFAT, FFQ_MFAT, FFQ_PFAT, FFQ_SFAT, FFQ_FIBE, FFQ_SUGR

Mortality codebook: Vital_Status, Vital_Status¬_Age, CauseOfDeath

CancerRegistry codebook: AgeDx, YEAR_DX, INCIDENCE_DAYS, BREAST_CANCER, COLORECTAL_CANCER, LUNG_CANCER, PROSTATE_CANCER, PRIMARY_SITE, SITE_GROUP

FollowUp1 codebook: AgeF1, DiabetesF1, Diabetes_AgeF1, Diabetes_MedF1, MIF1, MI_AgeF1, StrokeF1, Stroke_AgeF1, CancerF1, Cancer_AgeF1

FollowUp2 codebook: AgeF2, DiabetesF2, Diabetes_AgeF2, Diabetes_MedF2, MIF2, MI_AgeF2, StrokeF2, Stroke_AgeF2, CancerF2, Cancer_AgeF2

14. References:

(1) Kant AK. Dietary patterns and health outcomes. J Am Diet Assoc 2004;104:615-635

(2) Yu D, Zhang X, Xiang Y et al. Adherence to dietary guidelines and mortality: a report from prospective cohort studies of 134,000 Chinese men and women. (under review)

(3) Wirt A, Collins CE. Diet quality--what is it and does it matter? Public Health Nutr 2009;12:2473-2492.

(4) Guenther PM, Reedy J, Krebs-Smith SM. Development of the Healthy Eating Index-2005. J Am Diet Assoc 2008;108:1896-1901.

(5) Chiuve SE, Fung TT, Rimm EB et al. Alternative dietary indices both strongly predict risk of chronic disease. J Nutr 2012;142:1009-1018.

(6) McCullough ML, Feskanich D, Stampfer MJ et al. Diet quality and major chronic disease risk in men and women: moving toward improved dietary guidance. Am J Clin Nutr 2002;76:1261-1271.

(7) Belin RJ, Greenland P, Allison M et al. Diet quality and the risk of cardiovascular disease: the Women's Health Initiative (WHI). Am J Clin Nutr 2011;94:49-57.

(8) Reedy J, Mitrou PN, Krebs-Smith SM et al. Index-based dietary patterns and risk of colorectal cancer: the NIH-AARP Diet and Health Study. Am J Epidemiol 2008;168:38-48.

(9) Fung TT, Hu FB, McCullough ML, Newby PK, Willett WC, Holmes MD. Diet quality is associated with the risk of estrogen receptor-negative breast cancer in postmenopausal women. J Nutr 2006;136:466-472.

(10) Mursu J, Steffen LM, Meyer KA, Duprez D, Jacobs DR, Jr. Diet quality indexes and mortality in postmenopausal women: the Iowa Women's Health Study. Am J Clin Nutr 2013.

(11) Darmon N, Drewnowski A. Does social class predict diet quality? Am J Clin Nutr 2008;87:1107-1117.

(12) Hiza HA, Casavale KO, Guenther PM, Davis CA. Diet quality of Americans differs by age, sex, race/ethnicity, income, and education level. J Acad Nutr Diet 2013;113:297-306.

(13) Wang Y, Chen X. How much of racial/ethnic disparities in dietary intakes, exercise, and weight status can be explained by nutrition- and health-related psychosocial factors and socioeconomic status among US adults? J Am Diet Assoc 2011;111:1904-1911.

(14) Drewnowski A, Darmon N. The economics of obesity: dietary energy density and energy cost. Am J Clin Nutr 2005;82:265S-273S.

(15) Zamora D, Gordon-Larsen P, Jacobs DR, Jr., Popkin BM. Diet quality and weight gain among black and white young adults: the Coronary Artery Risk Development in Young Adults (CARDIA) Study (1985-2005). Am J Clin Nutr 2010;92:784-793.

(16) Kimokoti RW, Newby PK, Gona P et al. Diet quality, physical activity, smoking status, and weight fluctuation are associated with weight change in women and men. J Nutr 2010;140:1287-1293.

(17) Arem H, Reedy J, Sampson J et al. The Healthy Eating Index 2005 and Risk for Pancreatic Cancer in the NIH-AARP Study. J Natl Cancer Inst 2013;105:1298-1305.
